# Enhancing TB case detection: a case study of Kenya’s Global Fund–supported public–private mix

**DOI:** 10.5588/pha.26.0002

**Published:** 2026-03-06

**Authors:** R. Pola, L.N. Mugambi-Nyaboga, N. Mwirigi, A. Otieno, I. Kathure, N. Mukiri, S. Kipkelwon, A. Maina, P. Warugongo, C. Okoth, C. Mwamsidu, T. Kiptai, A. Munene, J. Mungai, J. Chakaya, E. Wandwalo, M.A Yassin, B. Ulo

**Affiliations:** 1 Public Health and Research Unit, Respiratory Society of Kenya, Nairobi, Kenya;; 2 Center for Health Solutions, Nairobi, Kenya;; 3 National TB Program, Ministry of Health, Nairobi, Kenya;; 4 County Government of Kiambu Department of Health, Kiambu, Kenya;; 5 Amref Health Africa, Nairobi, Kenya;; 6 The Global Fund to Fight Against HIV, TB and Malaria, Geneva, Switzerland.

**Keywords:** tuberculosis, case finding, private sector, notification

## Abstract

**BACKGROUND:**

Kenya, a high-TB-burden country, is among eight WHO-priority countries for public–private mix (PPM) initiatives to engage all health care providers in TB prevention and care.

**OBJECTIVE:**

To describe Kenya’s experience implementing a Global Fund–supported PPM intervention and its contribution to TB case finding.

**DESIGN:**

A descriptive case study using programmatic data from a Global Fund–supported PPM project implemented in nine counties in Kenya.

**RESULTS:**

Of 2,027 mapped facilities, 1,405 signed Memoranda of Understanding and 1,269 reported TB services. Of 4.3 million people screened, 260,922 (6%) were identified as presumptive TB, of whom 108,723 (42%) were investigated. Overall, 14,026 individuals were diagnosed with TB (64% bacteriologically confirmed), and 99% initiated on treatment. Level II facilities contributed 45% of notifications (7 per facility), while Level V facilities (only 4) reported the highest average yield (130 per facility). All counties recorded increased TB notifications during implementation, followed by a decline in Quarter 3, 2024.

**CONCLUSION:**

Engaging private sector providers significantly enhanced TB case detection. Kenya’s PPM experience highlights the engagement choices that need to be made among levels of the health care system for scaling and sustaining PPM models in resource-constrained settings.

TB remains a leading global cause of death from a single infectious agent.^1^ In 2024, an estimated 10.7 million people fell ill with TB, resulting in 1.23 million deaths.^2^ In Kenya, reported drug-susceptible TB cases increased from 77,854 in 2021 to 90,560 in 2022, a 16.7% increase attributed to intensified case-finding efforts.^3^ The 2016 Kenya national TB prevalence survey found a prevalence-to-notification ratio of about 2.5:1, indicating that only around 40% of people with TB were being captured by notification systems.^4^ Patient pathway analyses show that approximately 42% of people with presumptive TB initially seek care in the private sector, yet only 13%–21% of notified cases originate there.^5^ This disparity may be due to underdiagnosis, attrition along the care cascade, or under-reporting of diagnosed cases, underscoring the need for structured public–private mix (PPM) approaches.^6^ Globally, many patients seek care in the private sector: about half in sub-Saharan Africa, two thirds in Southeast Asia, and four fifths in South Asia, regardless of socio-economic status.^7^ Kenya mirrors this trend, as shown in the 2017 Patient Pathway analysis.^5^ Contributing factors to the private sector TB notification gap include weak integration with national programmes, limited provider training, and poor reporting mechanisms.^8^ Recognising this, the WHO endorsed PPM as a core strategy under the End TB Strategy, identifying Kenya among eight priority countries due to its high TB burden and reliance on private health care.^9^ Ensuring that private providers adhere to international standards of TB care is crucial to reducing transmission, providing quality services to people seeking care in the private sector, and preventing the spread of drug-resistant TB.^10–14^

Private providers are often preferred due to proximity, convenience, and extended service hours, making them essential partners in TB care.^15,16^ Through PPM, Kenya has increasingly involved private hospitals, clinics, faith-based organisations (FBOs), and chemists in TB service delivery. However, description of Kenya’s PPM experience has been limited. The first national comparison of TB care and prevention activities in public versus private facilities revealed programmatic challenges in private sector engagement.^17^ A recent Global Fund report emphasised the need to transition PPM financing to domestic resources for sustainability.^6^ Kenya’s health system is mixed: 52% of facilities are privately owned, including for-profit clinics, FBOs, and private hospitals, while 48% are public.^18^ Health service delivery sites in Kenya are categorised according to the available infrastructure and range of services, from level 1 at the community level to level 6, which includes national referral and teaching hospitals. The most common providers in both the public and private health sectors are level 2 facilities, which constitute 71% of all health facilities. Ownership and distribution of health facilities vary widely across counties,^19^ contributing to disparities in TB detection and reinforcing the need for structured private providers’ engagement.^20^ There is increasing evidence that the quality of TB care in the private sector falls short of international standards in many places.^21^ Many private facilities lack structured continuing education and reliable supervision, leading to variable adherence to national TB guidelines.^22^ At the same time, documentation and reporting remain weak: many private clinics struggle with consistent recording of presumptive cases and linking into national surveillance systems. These challenges contribute significantly to under-notification of TB in the private sector.^21^

With Global Fund support, Amref Health Africa and the Respiratory Society of Kenya implemented a PPM intervention in nine counties between 2021 and 2024. We describe the key processes and outcomes of private provider engagement in TB case finding and notification in Kenya. The findings provide insights for scaling PPM models nationally and contribute to global evidence on best practices for high-burden, low-resource settings.

## METHODS

This descriptive case study assessed the implementation and performance of Kenya’s PPM initiative for TB prevention and care supported by the Global Fund Grant Cycle 6 (2021–2024). The project was implemented from the third quarter of 2022 (July–September) through the second quarter of 2024 (April–June).

### Setting and population

Kenya has 47 counties organised into 12 health planning regions. Counties differ in population, TB burden, and health system capacity within a six-tier decentralised health system. The PPM initiative was implemented in private health facilities in nine counties across urban, peri-urban, and rural settings.

### PPM implementation approach

The intervention was led by Amref Health Africa, a non-state Principal Recipient of the Global Fund TB grant in Kenya, in partnership with the National Tuberculosis Program (NTP) and county health departments. The Respiratory Society of Kenya (ReSoK), a sub-recipient, oversaw implementation in nine counties, jointly selected with stakeholders based on TB burden, private sector presence, and readiness to engage. The PPM intervention included stakeholder engagement, private facility mapping, provider training, diagnostic referral linkages, and routine TB reporting. Detailed implementation procedures are described in Supplementary Data Appendix S1.

### Data collection and analysis

Data were obtained from routine project records and validated against the national TB database (TIBU). Key indicators included facility engagement, health care workers (HCWs) trained, TB diagnoses and treatment initiation, and number needed to screen (NNS). Descriptive statistics summarised performance across counties and facility types.

### Ethical statement

This study analysed routinely collected programme data. Ethical approval was not required as no individual patient-level data were used. All data were anonymised and aggregated before analysis. Oversight and approval were provided by the Ministry of Health through the NTP.

## RESULTS

Across the nine counties, 2,027 private health facilities were mapped (detailed facility engagement cascades are presented in Supplementary Data Table S1). Of these, 1,405 (70%) signed MoUs with county health departments, out of which 1,269 (90% of engaged facilities) reported services along the TB care cascade. Of the 1, 269 facilities reporting on the TB care cascade, there were 851 (67%) level 2, 152 (12%) level 3, 128 (10%) level 4, and 4 (0.3%) level 5 facilities and 112 (8%) chemists, 20 (1.5%) standalone laboratories, and 2(0.15%) imaging centres. Collectively, these represented 63% of all mapped facilities and 87% of those targeted.

### Capacity building

ReSoK conducted county-based trainings for HCWs from engaged private facilities. In total, 880 HCWs (77% of target) were trained.

### TB case-finding achievements

Across these facilities, 4.3 million individuals were symptomatically screened for TB. Of these, 260,922 (6%) were identified as people with presumptive TB, and 108,723 (42% of presumptive cases) underwent further diagnostic evaluation. In total, 14,026 individuals were diagnosed with TB; 64% were bacteriologically confirmed. Nearly all diagnosed patients (13,841; 99%) were initiated on treatment during the project period. Level II facilities, being the most numerous (n = 851), contributed 6,337 people with TB, representing 45% of all TB notifications from private providers. Although few in number, Level V facilities (n = 4) reported the highest average number of people with TB per facility (130 cases), followed by Level IV facilities (46 people with TB/facility), Level III facilities (8 people with TB/facility), and Level II facilities (7 people with TB/facility). Laboratories reported modest yields, while chemists and imaging centres contributed few or no confirmed cases (Table).

**TABLE.** TB case-finding achievement by facility type (Q3/2022–Q3/2024).

Facility type	Number of facilities	Total cases diagnosed	Average TB cases per facility
Chemist	112	38	0.3
Imaging	2	0	0
Lab	20	51	3
Level 2	851	6,337	7
Level 3	152	1,232	8
Level 4	128	5,849	46
Level 5	4	519	130
Total	1,269	14,026	11

### Number needed to screen (NNS) to find one person with TB

Overall, the NNS across private facilities was 308 (detailed NNS by facility type are presented in Supplementary Data Table S2). Higher-level facilities were more efficient, with the lowest NNS observed in Level V and IV facilities.

### Private sector contribution to county TB case finding

The Figure presents the contribution of the private sector to TB notifications from Q3 2022 to Q3 2024 (post-implementation). All counties recorded increased private sector contributions to TB notifications during implementation. A decline was noted in Q3 2024 across all counties except Meru, coinciding with the conclusion of project activities.

**FIGURE.**
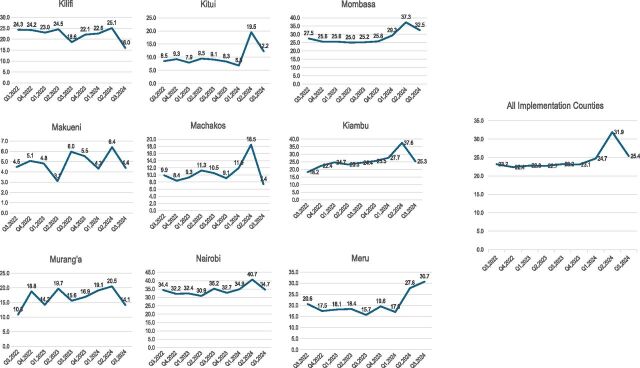
Private sector contribution to TB notification per quarter by county.

## DISCUSSION

The intervention achieved wide private facility engagement and contributed over 14,000 TB case notifications. These findings confirm that mobilising private providers can substantially strengthen national TB care and prevention in contexts where the private sector serves as a first point of care. Although Level II facilities contributed the largest volume of TB notifications, they were the least efficient, reflecting their large numbers, lower per-facility yield, and higher supervision needs. Level 2 private providers are important for early care seeking, yet they pose the greatest challenges in TB private provider engagement because of their large numbers, the relatively low case yield per provider, and low administrative capacities^21^ and the relatively high cost associated with their engagement, including training and supervisory needs. To effectively engage high proportions of these providers, more resources are required for the training, provision of technical support, and quality assurance.^23^ Higher-level facilities (Levels IV and V) were more efficient, with lower numbers needed to screen (NNS). Similar patterns have been reported in other high-burden settings.^24,25^ In addition, patients with more severe or prolonged symptoms tend to self-refer or be referred to higher-level hospitals, and the clinicians in these settings may have a higher index of suspicion for TB and greater experience recognising atypical presentations.^26^ Laboratories showed moderate efficiency, while pharmacies had high volumes but low diagnostic yield, underscoring the need for stronger referral linkages, consistent with recent studies from Bangladesh and Pakistan emphasising the need for stronger referral linkages and training.^27,28^

Importantly, private sector contributions to case finding increased across all counties during implementation, peaking at 37% in Kiambu and Mombasa. Similar gains have been reported in Ethiopia and India, where structured supervision and performance monitoring under PPM models significantly boosted notifications.^29,30^ The observed decline after project closure, however, underscores the fragility of donor-dependent gains and highlights the urgency of embedding private sector engagement within routine health system structures and domestic financing, an issue echoed in recent WHO and Stop TB recommendations.^31,32^ Training coverage for HCWs was lower than targeted in urban counties, where competing priorities may have limited provider availability. Emerging evidence suggests that flexible modalities, such as e-learning or off-peak in-person sessions, can improve provider engagement in such contexts.^19^ Future implementation should therefore tailor capacity-building strategies to urban provider dynamics while sustaining engagement in rural and peri-urban areas.

Kenya’s experience demonstrates that structured PPM models can substantially expand TB case detection but require integration into routine health systems for sustainability. These findings contribute to global evidence on the feasibility and effectiveness of PPM models.

## CONCLUSION

This case study demonstrates that structured engagement of private providers through a PPM model can significantly expand TB case detection and notification in Kenya. By mobilising diverse providers and integrating them into national reporting systems, over 14,000 additional TB cases were identified across nine counties. While level 2 facilities contributed the highest number of notifications, they were the least efficient, highlighting the choices that need to be made when engaging levels of the health care system for scaling and sustaining PPM models in resource-constrained settings.

## Supplementary Material




