# Supplementary material for: Enhancing TB case detection: a case study of Kenya’s Global Fund–supported public–private mix
Source: Public Health Action. 2026 Mar 6;16(1):59–62. doi: 10.5588/pha.26.0002 (PMC12991482; doi:10.5588/pha.26.0002)
Supplement: Supplementary file 1 [file pha26-0002_supplementarydata1.pdf]

Supplementary Appendix

Supplementary Appendix S1

Detailed Description of the Public–Public–Private Mix (PPM) Intervention and Method

1. Study Context and Setting

Kenya is a high tuberculosis (TB) burden country with a mixed public–private health system. TB services are coordinated nationally by the National Tuberculosis Program (NTP) within a decentralized six-tier health system. Private sector providers, including for-profit clinics, faith-based organizations, stand-alone laboratories, imaging centres, and pharmacies, constitute over half of all registered health facilities in the country.

The Global Fund–supported PPM intervention was implemented between 2021 and 2024 in nine counties selected based on TB burden, density of private providers, and readiness for engagement. These counties represented urban, peri-urban, and rural contexts.

2. PPM Implementation Framework

2.1 Governance and Partnerships

The PPM intervention was led by Amref Health Africa as Principal Recipient of the Global Fund TB grant, in collaboration with the National Tuberculosis Program and county health departments. The Respiratory Society of Kenya (ReSoK) served as a sub-recipient responsible for implementation oversight in the nine counties.

County TB leadership provided stewardship, facilitated private sector entry, and ensured alignment with routine TB program structures.

3. Pre-Implementation Activities

3.1 Stakeholder Engagement

Stakeholder entry meetings were conducted in all participating counties to introduce the PPM approach, clarify roles, and align implementation with county TB priorities. Participants included county TB coordinators, sub-county teams, professional associations, and representatives of private providers.

3.2 Facility Mapping and Readiness Assessment

A digital mapping exercise was conducted to identify all private health facilities in the nine counties. A structured assessment tool was used to capture facility characteristics, service capacity, diagnostic access, staffing, and prior engagement in TB services.

Facilities expressing willingness to participate signed Memoranda of Understanding (MOUs) with county health departments, outlining responsibilities related to TB screening, referral, diagnosis, treatment linkage, and reporting.

4. Capacity Building and Supportive Supervision

4.1 Training of Healthcare Workers

Targeted trainings were conducted for clinicians, laboratory personnel, and facility managers from engaged private facilities. The curriculum covered:

- TB screening and diagnosis (including drug-resistant TB),
- infection prevention and control,
- TB preventive therapy,
- treatment initiation and follow-up, and
- documentation and reporting using national tools.

Laboratory staff received refresher training on sputum smear microscopy and quality assurance.

4.2 Supportive Supervision

Routine supportive supervision visits were conducted jointly by county TB teams and implementing partners to reinforce adherence to national TB guidelines, address reporting gaps, and provide on-site mentorship.

5. Diagnostic Referral and Sample Transport

A hub-and-spoke model was used to facilitate diagnostic access for private facilities without on-site molecular testing. Peripheral facilities referred specimens to GeneXpert hubs located in public or higher-level private facilities.

Specimen transport was supported through motorcycle couriers. Modest incentives were provided to hub laboratories to offset additional workload associated with processing referred samples.

6. Tuberculosis Diagnostic Procedures

All diagnostic investigations were performed according to national TB guidelines.

6.1 Pulmonary TB

For presumptive pulmonary TB, sputum samples were tested using GeneXpert MTB/RIF or MTB/RIF Ultra where available. In facilities without GeneXpert capacity, smear microscopy was performed, with referral for molecular testing when feasible.

6.2 Extrapulmonary TB

For presumptive extrapulmonary TB, appropriate specimens (e.g., lymph node aspirates, pleural fluid, biopsy samples) were collected and processed using cytological, histopathological, or molecular methods as available.

7. Treatment Initiation and Linkage to Care

Patients diagnosed with TB through private facilities were initiated on treatment either within designated private facilities or referred to nearby public TB treatment centers, depending on county arrangements. Treatment initiation and outcomes were recorded in national TB registers.

8. Data Sources and Management

Data were obtained from routine project tools, including:

- facility engagement logs,
- training registers,
- monthly TB screening and notification reports, and
- county review meeting reports.

Notification data were cross-validated against the national electronic TB database (TIBU) to ensure completeness and consistency.

9. Outcome Measures and Analysis

Key indicators included:

- proportion of mapped private facilities engaged and reporting TB services,
- number of healthcare workers trained,
- number of people screened, presumptive TB cases identified, and TB cases diagnosed,
- treatment initiation rates, and
- Number Needed to Screen (NNS).

Descriptive analyses were conducted to summarize outputs across counties and facility types. Facility-level efficiency was assessed using NNS, calculated as the number of individuals screened divided by the number of TB cases diagnosed.

Supplementary Tables

Supplementary Table S1: Cascade of private facility engagement from Mapping to Reporting

County	Facilities Mapped	Number targeted	Declined	Closed	Total engage	Signed MoU	Facilities Reporting	% Achievement<br><br>(Signed MoU Vs. Reporting)
Kiambu	318	249	52	3	263	217	173	80%
Murang'a	133	89	11	3	119	114	95	83%
Meru	282	152	18	14	250	192	162	84%
Kilifi	124	121	11	3	110	104	103	99%
Mombasa	189	181	46	6	137	126	106	84%
Machakos	182	102	0	2	180	149	149	100%
Makueni	93	60	1	1	92	90	90	100%
Kitui	99	65	2	3	97	97	97	100%
Nairobi	607	447	98	60	449	316	294	93%
Total	2027	1466	239	95	1694	1405	1269	90%

Supplementary Table S2: Number Needed to Screen (NNS) to diagnose one TB case by facility type

Type of facility	Total people screened for TB	TB cases diagnosed	NNS
Chemist	11,624	38	306
Imaging site	48	-	-
Laboratory	10,599	51	208
Level 2	2,010,690	6,337	317
Level 3	721,746	1,232	586
Level 4	1,529,233	5,849	261
Level 5	35,769	519	69
Total	4,319,709	14,026	308
